# *Plasmodium falciparum* phenotypic and genotypic resistance profile during the emergence of Piperaquine resistance in Northeastern Thailand

**DOI:** 10.1038/s41598-021-92735-6

**Published:** 2021-06-28

**Authors:** Nonlawat Boonyalai, Chatchadaporn Thamnurak, Piyaporn Sai-ngam, Winita Ta-aksorn, Montri Arsanok, Nichapat Uthaimongkol, Siratchana Sundrakes, Sorayut Chattrakarn, Chaiyaporn Chaisatit, Chantida Praditpol, Watcharintorn Fagnark, Kirakarn Kirativanich, Suwanna Chaorattanakawee, Pattaraporn Vanachayangkul, Paphavee Lertsethtakarn, Panita Gosi, Darunee Utainnam, Wuttikon Rodkvamtook, Worachet Kuntawunginn, Brian A. Vesely, Michele D. Spring, Mark M. Fukuda, Charlotte Lanteri, Douglas Walsh, David L. Saunders, Philip L. Smith, Mariusz Wojnarski, Narongrid Sirisopana, Norman C. Waters, Krisada Jongsakul, Jariyanart Gaywee

**Affiliations:** 1 Department of Bacterial and Parasitic Diseases, Armed Forces Research Institute of Medical Sciences, Bangkok, Thailand; 2 Department of Parasitology and Entomology, Faculty of Public Health, Mahidol University, Bangkok, Thailand; 3 Department of Retrovirology, Armed Forces Research Institute of Medical Sciences, Bangkok, Thailand; 4 U.S. Army Research Institute of Infectious Diseases, Frederick, MD USA; 5 Walter Reed Army Institute of Research, Silver Spring, MD 20910 USA; 6 Royal Thai Army Component, Armed Forces Research Institute of Medical Sciences, Bangkok, Thailand; 7 Department of Dermatology, Syracuse VA medical center, Syracuse, USA

**Keywords:** Malaria, Parasite genetics

## Abstract

Malaria remains a public health problem in Thailand, especially along its borders where highly mobile populations can contribute to persistent transmission. This study aimed to determine resistant genotypes and phenotypes of 112 *Plasmodium falciparum* isolates from patients along the Thai-Cambodia border during 2013–2015. The majority of parasites harbored a *pfmdr1*-Y184F mutation. A single *pfmdr1* copy number had CVIET haplotype of amino acids 72–76 of *pfcrt* and no *pfcytb* mutations. All isolates had a single *pfk13* point mutation (R539T, R539I, or C580Y), and increased % survival in the ring-stage survival assay (except for R539I). Multiple copies of *pfpm2* and *pfcrt*-F145I were detected in 2014 (12.8%) and increased to 30.4% in 2015. Parasites containing either multiple *pfpm2* copies with and without *pfcrt*-F145I or a single *pfpm2* copy with *pfcrt*-F145I exhibited elevated IC_90_ values of piperaquine. Collectively, the emergence of these resistance patterns in Thailand near Cambodia border mirrored the reports of dihydroartemisinin-piperaquine treatment failures in the adjacent province of Cambodia, Oddar Meanchey, suggesting a migration of parasites across the border. As malaria elimination efforts ramp up in Southeast Asia, host nations militaries and other groups in border regions need to coordinate the proposed interventions.

## Introduction

An estimate of 229 million malaria cases occurred worldwide in 2019^1^ and artemisinin-based combination therapies (ACT) have been the first-line drug treatment for uncomplicated *Plasmodium falciparum* infection for most malaria endemic areas; however, resistance to both artemisinin (ART) and its partner drugs has increased over the years at a pace requiring intensive surveillance and monitoring. The first case reports of ART resistance emerged in Thailand in the late 1990s^2^ and in 2003–2005. In 2008, Thai Ministry of Public Health (MoPH) and Armed Forces Research Institute of Medical Sciences (AFRIMS) studies reported that first-line ACT regimens (2-day artesunate (AS) plus mefloquine (MQ)) were failing along both sides of the Thai-Cambodia border near Trat, Thailand and Pailin, Cambodia^3^. Militaries and other mobile forest goers in these border areas constitute a population at risk for succumbing to multi-drug resistant malaria as well as facilitating transmission, necessitating targeted control strategies if the Greater Mekong Subregion (GMS) is to achieve malaria elimination.

Following the case reports of ACT failure, the Artemisinin Resistance: Confirmation, Characterization and Planning for Containment Project (ARC) in 2006 showed a delayed clearance resistance phenotype in Cambodia along the Thai border which continued to spread across mainland Southeast Asia^4–9^. It was not until 2014 that an ART resistance molecular marker was identified with mutations in the propeller domain of *P. falciparum* Kelch-13 gene (*pfk13*) (PF3D7_1343700)^10–13^, which can be confirmed by the ring-stage survival assay (RSA)^10,14^ and/or delayed parasite clearance times in clinical trials^4,5^. Multiple copies of *P. falciparum* multi drug resistance 1 (*pfmdr1*) (PF3DF_0523000) is a well-established marker for MQ resistance^15,16^, while the *pfmdr1* single-nucleotide polymorphisms (SNPs) have been associated with modulation of parasite tolerance or susceptibility to several antimalarial drugs including quinine (QN), amodiaquine (AQ), chloroquine (CQ), MQ and lumefantrine (LUM)^17^. The main *pfmdr1* SNPs associated with drug resistance include N86Y, Y184F, S1034C, and N1024D^18–23^. Mutation N86Y in *pfmdr1* is associated with increased CQ resistance but increased sensitivity to MQ^24^. The *pfmdr1* 184F mutation alone was not associated with susceptibility response of CQ, MQ, LUM, QN, monodesethylamodiaquine (MDAQ), and dihydroartemisinin (DHA)^25^. However, the parasites carrying *pfmdr1* 86Y and Y184 haplotype was associated with increased susceptibility to MDAQ, LUM, and MQ. The possible role of *pfmdr1* N86, 184F and 1246D alleles and *pfmdr1* copy number associated with artemether-lumefantrine resistance is not confrmed^26^. An ACT alternative to AS-MQ is dihydroartemisinin-piperaquine (DHA-PIP) and the first PIP resistance markers identified were multiple copies of *P. falciparum* plasmepsin 2 (*pfpm2*) (PF3D7_1408000)^27–30^, followed by mutations in the *P. falciparum* chloroquine resistance transporter (*pfcrt*) (PF3D7_0709000) downstream of the 4-aminoquinoline resistance locus (positions 72–76 with K76T)^31–34^, and the E415G mutation in *P. falciparum* exonuclease (*pfexo*) (PF3D7_1362500)^27,28,35^. PIP resistance can be characterized by the elevated IC_90_ values^36^ and PIP survival assay (PSA) with a survival rate of more than 10%, defining a PIP resistant phenotype^31^. Atovaquone-proguanil (ATQ-PG) is not an ACT but ATQ resistance subsequently emerged, which was linked to specific mutations in *P. falciparum* mitochondrial cytochrome B (*pfcytb*) (AY282930.1) in particular the mutation at positions 258 and 268^37–39^.

Efforts in mapping parasite population structure and gene flow can assist in understanding and predicting the spreading pattern of resistance. Military populations at border areas who are deployed to endemic areas should be integrated in surveillance and monitoring efforts as they are on the front-lines of malaria transmission. Here we report resistance characteristics of *P. falciparum* clinical isolates collected from Thai soldiers and civilian patients between 2013 and 2015 and show the trends in drug resistance. At that time, the national treatment guidelines in Thailand recommended AS-MQ for *P. falciparum* malaria. During this same period, we reported dramatic loss of efficacy of DHA-PIP (54% treatment failure) in a study that was conducted by AFRIMS in Anlong Veng, 12 km from the Thai border of Sisaket Province^40^. Intensive malaria surveillance to track drug resistance is required in high risk military and other border populations to achieve malaria control.

## Materials and methods

### Study setting, protocol and subjects

This minimal risk surveillance study was open between July 2013-September 2015, enrolling Thai adults (aged 18 years and over) presenting with uncomplicated *P. falciparum* or *P. falciparum*/*P. vivax* mixed-infections at military health facilities. The enrollment population included soldiers, border police, or their family members and other villagers located near the Royal Thai Army (RTA) health clinics in Sisaket and Surin Provinces, located in northeastern Thailand on the Thai-Cambodia border. Inclusion criteria were asexual parasitemia per rapid diagnostic test (RDT) or blood film, and no malaria infection or antimalarials taken within the past seven days. The protocol was approved by the Walter Reed Army Institute of Research Institutional Board, Institute for Development of Human Research Protection, and RTA Institutional Review Board. All research was performed in accordance with relevant guidelines and regulations. All study subjects provided written informed consent prior to participation. Goal enrollment was 50 malaria cases per year, balancing the number of isolates needed to characterize resistant genotypes/phenotypes with the local population size and expected incidence of cases that could be enrolled. While treatment was uniformly provided per national guidelines at the time, volunteers were not followed up for treatment efficacy.

### Sample collection and preparation

Patients diagnosed with *P. falciparum* infection at RTA clinics were subjected to peripheral venipuncture prior to treatment. Two microscopists examined Giemsa-stained peripheral blood smears for each volunteer to determine malaria species infection and parasite densities for blood stages. Venous blood samples were collected in EDTA tubes for DNA extraction and molecular characterization and in sodium-heparin tubes for ex vivo bioassay and in vitro drug sensitivity assay. Plasma was separated from blood, frozen at − 80 °C, and infected packed red blood cells were cryopreserved. All blood and processed blood samples were stored at − 80 °C and transported in dry ice to AFRIMS for molecular characterization, ex vivo bioassay, and in vitro culture adaptation and drug sensitivity testing.

### Molecular markers of malaria drug resistance

Parasite genomic DNA was extracted from 200 µL of EDTA whole blood by using EZ1 DNA blood kits with an automated EZ1 Advanced XL purification system as per the manufacturer’s instructions. The DNA was stored at − 20 °C. T100TM Thermal Cycler (Bio-Rad Laboratories) was employed to evaluate resistance makers including, *P. falciparum* kelch13 propeller domain (*pfk13*) (PF3D7_1343700), *pfcrt* SNP F145I (KM288867.1), and *pfcytb* SNPs (AY282930.1)^10,41^. ABI 7500 Real-time PCR system (Applied Biosystems) was employed to characterize *pfcrt* SNPs (72–76) and *pfmdr1* SNPs (Positions 86, 184, 1034, 1042). Primers used to identify *pfkelch13*, *pfcytb*, *pfcrt* and *pfmdr1* SNPs are shown in Tables S1 and S2.

### Copy number variation assay

To determine copy numbers of *pfmdr1* and *pfpm2* genes, real-time quantitative PCR (qPCR) was performed on genomic DNA as previously described^15,29,42^ with some modifications. For *pfmdr1*, amplification reactions were performed according to the TaqMan Real-time PCR methods using ABI 7500 Real-time PCR system (Applied Biosystems) with 200 nM of each forward and reverse primer (Table S2) and 2 ng of DNA template while Rotor-Gene Q (QIAGEN, Valencia, CA) using Type-it® HRM™ kit was employed for *pfpm2*^29^. The primers and probes (Table S2) used were as previously described to amplify the following loci: *pfmdr1* (PF3D7_0523000) and *pfpm2* (PF3D7_1408000), respectively^29^. For the housekeeping gene, β*-tubulin* (PF3D7_1008700), β-tubulin forward and reverse primers were designed and used as a reference control for all experiments with the same validated PCR conditions as target primers. *P. falciparum* 3D7 and Dd2 were used as references for single and multiple copy numbers of *pfmdr1*, respectively. All samples including the references clones were performed in duplicate. The average copy number values for each genes were calculated using 2^−ΔΔCt^ method. Parasites with copy number greater than 1.5 copies for *pfmdr1*^15^ and 1.6 copies for *pfpm*2^29^ were interpreted to contain multiple copies of each gene.

### In vitro culture adaptation and maintenance of parasites

Of 112 collected samples, 86 samples were attempted for in vitro culture adaptation. The exclusion criteria of the in vitro culture adaptation were *P. falciparum/P. vivax* mixed infections, % parasitemia < 0.05, and ex vivo bioassay activity > 250 nM (DHA equivalent). Culture adaptation of the parasites was performed using the modification method of Trager and Jensen^43^. Parasites were maintained in fresh human erythrocytes (O^+^) in RPMI-1640 medium (Sigma), containing 15% AB^+^ human serum (heat inactivated and pooled) and supplemented with 25 mM HEPES, 25 mM sodium bicarbonate, and 0.1 mg/mL gentamicin. Human blood products (erythrocytes and serum) were obtained from the Thai Red Cross. Culture flasks were gassed with 5% CO_2_, 5% O_2_, 90% N_2_ and incubated at 37 °C. After culture adaptation the cultured parasites were cultivated for 3 cycles until enough material was obtained and they were synchronized with 5% D-sorbitol (Sigma) to obtain at least 70% ring forms before in vitro assays were run.

### In vitro 72 h drug susceptibility by Histidine rich protein 2 (HRP2)

Drug susceptibility test using HRP2-ELISA to measure 50% or 90% inhibitory concentration (IC_50_ and IC_90_) was performed following previously published methods^44–46^. Dried drug-coated plates containing antimalarial drugs as described in Chaorattanakawee et al.^36,45^ were used and in vitro drug susceptibility testing was carried out for control reference clones (W2, D6, C2B) (Malaria Research & Reference Reagent Resource, Manassas, Vermont, USA) as described previously^36^. IC_50_s and IC_90_s were estimated by nonlinear regression analysis using GraphPad Prism version 6.0.

### Ring-stage survival assay (RSA)

In vitro RSA_0-3 h_ was performed on 0–3 h post-invasion rings obtained from selected culture-adapted clinical isolates following published methods with slight modifications^14,46^. Parasite cultures were synchronized using 5% D-sorbitol and 75% Percoll to obtain 0 to 3-h post-invasion rings which were adjusted to 0.5–1% starting parasitemia with a 2% hematocrit in culture media (0.5% Albumax RPMI 1640 with 2.5% AB serum), and cultured in a 48-well microplates with 700 nM DHA and 0.1% DMSO in separate wells. The culture plates were then incubated for 6 h at 37 °C in modular incubator chambers and gassed with 5% CO_2_, 5% O_2_ and 90% N_2_. Cells were then washed once, resuspended in a drug-free medium, and cultured further for 66 h. Susceptibility to DHA was assessed microscopically on thin films by estimating the percentage of viable parasites, relative to control (% survival rate). For the controls, the RSA_0–3 h_ was also performed on *P. falciparum* reference clones W2, IPC-4884 and IPC-5202 (BEI Resources, NIAID, NIH, USA). A survival rate > 1% was deemed resistant for RSA.

### Ex vivo bioassay

*P. falciparum*-based bioassay was carried out to measure the antimalarial activity of patient plasma identifying if study volunteers were likely to have recently taken antimalarial drugs. Plasma was prepared from blood collected on the screening day for evaluation according to previously published methods^47,48^. In brief, the complement-inactivated samples were serially diluted and applied in one column to 96-well microplate at 50 µL/well. Two columns with serial dilutions of spiked plasma were added to each plate as controls. In addition to the plates with unknown samples, one plate was dosed with six serial dilutions in duplicate of DHA (from 100 to 2.5 ng/mL). A suspension of 200 µL of malaria parasite-infected red blood cells (W2 clone; 0.05% parasitemia with > 80% rings at a 1.7% hematocrit) was added to all wells. The microplates were placed into a chamber, flushed with a mixture of gas consisting of 5% CO_2_, 5% O_2_ and 90% N_2_, and plated into an incubator at 37 °C for 72 h. HRP2-ELISA was performed as described above.

### Liquid chromatography-tandem mass spectrometry (LC–MS/MS) analysis

To detect baseline antimalarials prior to treatment in the study population, plasma samples were extracted by using protein precipitation twofold volume of acetonitrile containing internal standard, then 1-min vortex mixing, 10 min-centrifugation, filtration of supernatant with a 0.22 µm PTFE filter and then transferred to an HPLC vial. The LC separation was performed on ACQUITY UPLC (Waters) coupled with tandem mass spectrometer (Xevo TQ-S, Waters) and eluted on Waters Acquity UPLC® BEH C18, 2.1 × 50 mm, 1.7 µm column at a flow rate of 0.5 mL/minute in 8 min run time. Mobile phase consisted of (A) 5 mM ammonium acetate pH 4.5 in water and (B) 5 mM ammonium acetate pH 4.5 in acetonitrile:methanol (50:50 v/v). The gradient started with 10% B, raised to 98% B in 6 min and held at this composition for 30 s, decreased to 10% B and re-equilibrated for 1 min. Column temperature was set at 40 °C. Selective mass to charge (m/z) transition for each compound was monitored as follow: AS (407.4 > 261.23), DHA (307.13 > 261.26), MQ (379.1 > 321.0), carboxy primaquine (cMQ) (310.22 > 290.05), PG (256.1 > 171.9), cycloguanil (CYC) (251.95 > 194.9), primaquine (PQ) (260.07 > 85.95), carboxy primaquine (cPQ) (275.34 > 175.11), orthoquinone (OQ) (260.2 > 147.08), stable isotopic labeled artesunate (SIL-AS) (411.3 > 261.18), and stable isotopic labeled primaquine (SIL-PQ) (264.35 > 86.15).

### Statistical analysis

Statistical analysis was performed using GraphPad Prism version 6.0 (GraphPad Software, Inc., San Diego, CA, USA). The difference of data between groups was assessed by nonparametric Kruskal–Wallis, Mann–Whitney or Dunn’s multiple comparison tests, as appropriate. Statistical significance was defined as a *P* value < 0.05.

### Ethics approval and consent to participate

All participants or guardians provided written consent and samples were collected under approval Royal Thai Army Institutional Review Board (RTA IRB) and Walter Reed Army Institute of Research Institutional Review board.

### Disclaimer

Material has been reviewed by the Walter Reed Army Institute of Research. There is no objection to its presentation/publication. The opinions or assertions contained herein are the private views of the author, and are not to be construed as official, or as reflecting true views of the Department of the Army or the Department of Defense. The investigators have adhered to the policies for protection of human subjects as prescribed in AR 70–25.

## Results

### Study population, demographic, and parasitological parameters

In total, 117 individuals were enrolled but 5 individuals were excluded from analysis due to lack of *P. falciparum* on a blood smear in 4 patients, and *P. vivax* monoinfection in 1 patient. Therefore, 112 individuals with uncomplicated *P. falciparum* were included in the analysis (Table 1). The median age of the participants was 23 years (IQR: 22–30.75) and most were male (98.2%), in the military (83%), and from Sisaket Province (96.4%). Among rapid diagnostic test and/or microscopy positive malarial isolates, 108 samples (96.4%) were confirmed by PCR as *P. falciparum* infections and 4 samples (3.6%) as mixed *P. falciparum* and *P. vivax* infections. The geometric mean of parasitemia of the participants was 12,097 parasites/µL.

**Table 1:** Patient and parasitological characteristics of 112 participants.

	**2013**	**2014**	**2015**	***P value***
Number of cases	27	39	46	–
Age (y), median (IQR)	22 (22–24)	23 (22–31)	23 (22–44)	0.1210
Male:female, n (%)	27:0 (100:0)	39:0 (100:0)	44:2 (96:4)	–
Civilian:Military, n (%)	1:26 (4:96)	5:34 (13:87)	13:33 (28:72)	–
Weight (kg), median (IQR)	60 (56–67)	62 (60–71)	60 (58–67)	0.698
Site location, Sisaket:Surin, n (%)	27:0 (100:0)	35:4 (90:10)	46:0 (100:0)	–
Parasitemia (no./µL), geometric mean (95% CI)	11,963 (6,698–17,944)	14, 960 (8,940–25,035)	10,704 (7,158–16,008)	0.320
*P. falciparum*, n (%)	27 (100%)	36 (92%)	45 (98%)	–
Mixed *P. falciparum*/*P. vivax*, n (%)	0	3 (8%)	1 (2%)	–

*P values* calculated by the Kruskal–Wallis test.

### Mutations in *pfmdr1*, *pfcrt, pfk13*, and *pfcytb* gene

112 samples were evaluated for SNPs of *pfmdr1*, *pfcrt* (positions 72–76), *pfcrt* (position 145), *pfk13*, and *pfcytb* as molecular markers of drug resistance associated with MQ, CQ, PIP, ART, and ATQ, respectively. Of four point mutations on *pfmdr1*, only the Y184F mutation (97.3%) was detected (Table 2), suggesting the presence of two *pfmdr1* haplotypes in the samples: NYSN and NFSN (representing amino acids positions 86, 184, 1034, and 1042, respectively) with NFSN as the most prevalent genotype. Genotyping of *pfcrt* showed all mutant haplotypes at positions 72–76 (CVIET), which is associated with CQ resistance. Regarding the novel *pfcrt* mutation (F145I) associated with PIP resistance, no F145I mutation was identified in 2013, but the F145I mutation of *pfcrt* was detected in 5 (12.8%) samples and 14 (30.4%) in 2014 and 2015, respectively. Concerning *pfk13* as associated with ART resistance, one of the three nonsynonymous mutations (R539T, R539I, or C580Y) in the *k13* propeller domain was observed in 112 samples and C580Y was the predominant *pfk13* mutation, accounted for 84.8% of all *pfk13* mutations. The number of C580Y mutants increased from 2013 (63%) to 2015 (100%). There were no mutations detected in *pfcytb* for amino acid positions 258 and 268.

**Table 2:** Mutations of malaria resistance molecular markers of *P. falciparum* in Thailand between 2013 and 2015.

Year	No. of tested	***pfmdr1***				***pfcrt***		***pfk13***			***pfcytb***	
		N86Y	Y184F	S1034C	N1042D	CVIET (72–76)	F145I	R539T	R539I	C580Y	I258M	Y268S
2013	27	0	27 (100%)	0	0	27 (100%)	0	9 (33.3%)	1 (3.7%)	17 (63.0%)	0	0
2014	39	0	36 (92.3%)	0	0	39 (100%)	5 (12.8%)	7 (17.9%)	0	32 (82.1%)	0	0
2015	46	0	46 (100%)	0	0	46 (100%)	14 (30.4%	0	0	46 (100%)	0	0
Total	112	0	109 (97.3%)	0	0	112 (100%)	19 (16.9%)	16 (14.3%)	1 (0.9%)	95 (84.8%)	0	0

Year	No. of tested	***pfmdr1* CNV**		***pfpm2* CNV**								
		Single copy	Multiple copy (> 1.5)	Single copy	Multiple copy (> 1.6)							
2013	27	24 (88.9%)	3 (11.1%)	25 (92.6%)	2 (7.4%)							
2014	39	37 (94.8%)	2 (5.2%)	19 (48.7%)	20 (51.3%)							
2015	46	46 (100%)	0	10 (21.7%)	36 (78.3%)							
Total	112	107 (95.5%)	5 (4.5%)	54 (48.2%)	58 (51.8)							

### Copy number variation of *pfmdr1* and *pfpm2*

Single *pfmdr1* copies were present in nearly all of evaluable cases (107/112), only 5 samples had multiple copies of *pfmdr1*, found in samples collected in 2013 and 2014. Multiple *pfpm2* copies were found in about half of evaluable cases (58/112), and the number of parasites harboring multiple *pfpm2* copies steadily rose from 7.4 to 78.3% from 2013 to 2015. Of 5 samples containing multiple *pfmdr1* copies, all were single copy *pfpm2* with *pfcrt* F145 wild-type. All parasites harboring multiple *pfpm2* copies contained the *pfpk13*-C580Y mutation and of those samples, 13 (22.4%) contained the novel *pfcrt* F145I mutation.

### Haplotype and copy number variation (CNV) of *P. falciparum* isolates

The parasite isolates were categorized into nine groups according to their genotypes of *pfmdr1*, *pfk13*, and *pfcrt* in combination with their CNV of *pfmdr1* and *pfpm2* (Table S3). Overall, the most prevalent parasites were those in group III, containing *pfk13*-580Y alleles, multiple *pfpm2* copies, *pfcrt*-F145 alleles and *pfmdr1* 184F alleles with a single copy number. This was followed by parasites in group II which was the same as Group III except with *pfpm2* single copy number.

The number of parasites in group II decreased from 55.6% to 13%, while those in group III increased from 7.4 to 56.5% over time. Parasites in group VI, containing the *pfcrt*-145I and multiple *pfpm2* copies, were also found to be increasing over the study period. It was noted that parasites in group VII to IX, harboring the *pfk13*-539I/T alleles with no novel mutation on *pfcrt*, and *pfmdr1-*184F only held a single *pfpm2* copy.

### Prevalence of molecular markers for ART-, MQ- and PIP-Resistance

With limited number of ACT options, we assessed if any parasites had all mutations for ART-, MQ-, and PIP-resistance (*pfk13* SNPs, *pfmdr1* CNV, *pfpm2* CNV, and *pfcrt-*F145I mutation). No tested parasites in this study carried all the aforementioned markers, although this is largely driven by having only 5 isolates with multiple copies of *pfmdr1*. If that marker is excluded, only 11.6% of the isolates (13/112) harbored *pfk13*-C580Y, multiple *pfpm2* copies, and *pfcrt*-F145I (3 isolates in 2014 and 10 isolates in 2015). All of the 16 parasites with *pfk13*-R539T carried a single *pfpm2* copy number with no *pfcrt*-F145I mutation but four of them had multiple *pfmdr1* copies.

**Figure 1:**
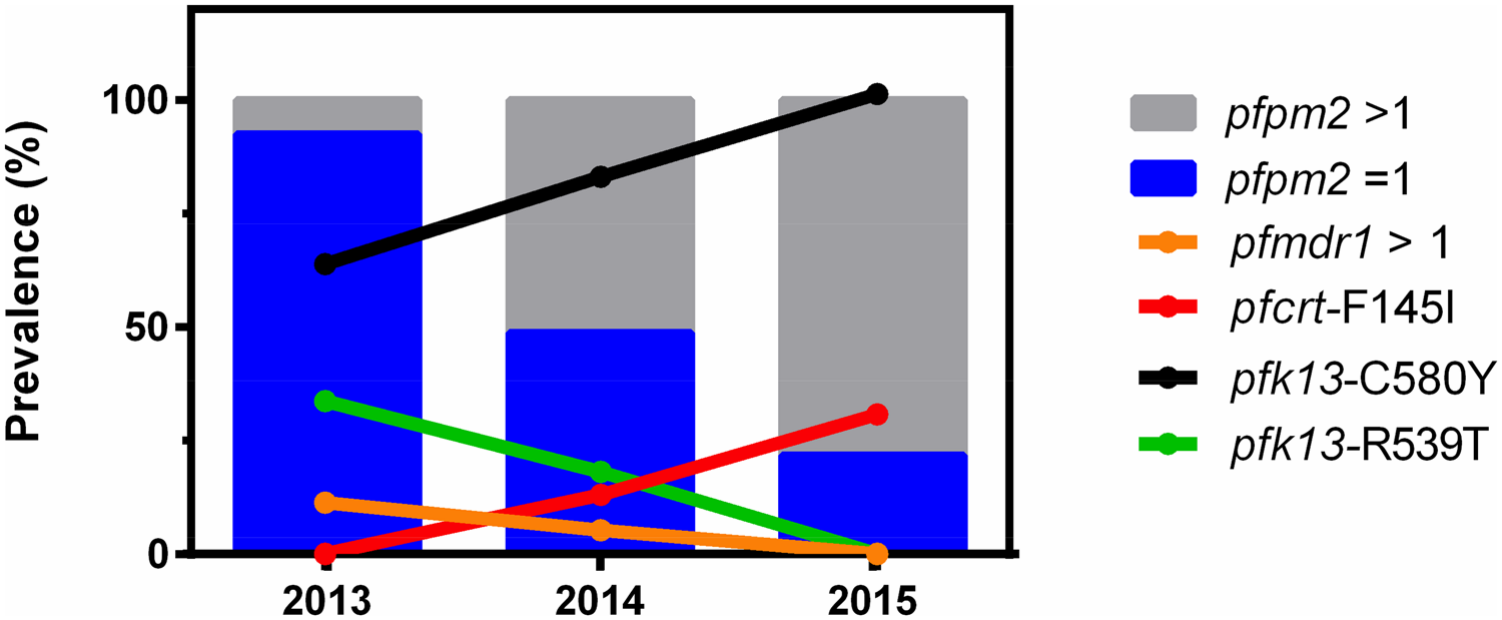
Prevalence of antimalarial drug resistance mutations in Thailand from 2013–2015. Bars indicate the prevalence of parasites with single and amplified *pfpm2* copy number. The line graphs indicate the prevalence of parasites harboring the *pfcrt-*F145I mutation, amplified *pfmdr1* copy number and *pfk13*-C580Y and *pfk13*-R539T mutations.

Figure 1 shows the prevalence of *pfpm2* copy number variation, *pfmdr1* copy number variation, *pfcrt*-F145I, *pfk13*-C580Y and *pfk13*-R539T mutations over time. The prevalence of parasites with multiple *pfpm2* copies associated with PIP resistance increased from 2013 to 2015, similar to the prevalence of parasites harboring *pfcrt*-F145I and *pfk13*-C580Y mutations. In contrast, the prevalence of parasites with multiple *pfmdr1* copies associated with MQ resistance and *pfk13*-R539T mutation decreased after 2013 through 2015.

### In vitro drug susceptibility of *P. falciparum* isolates

Of 86 *P. falciparum* monoinfection samples that met the parasitemia level criteria for in vitro culture, in vitro drug susceptibility of only 40 samples (11, 11 and 18 samples from 2013, 2014, and 2015, respectively) against an antimalarial drug panel could be obtained by HRP2-ELISA (Table 3). In viewing the geometric mean (GM) IC_50_ data for all drugs by year, CQ, PIP, CYC, MQ, and QN all exhibited statistically significant decreases in drug susceptibility. Compared to the ART- and MQ-sensitive W2 reference clone, isolates had greatly reduced sensitivity to PIP, CYC, and DOX, with PIP and CYC having a noticeable drop in effectiveness between 2014 and 2015. In contrast, while there was a steady decline in CQ and MQ sensitivity from 2013 to 2015 the GM-IC_50_ values remained lower than that of the CQ-resistance W2 reference (IC_50_-CQ = 234 nM) and MQ-resistance D6 reference (IC_50_-MQ = 118 nM). There was a modest increase in DHA and AS susceptibility, which did not change dramatically over the 3-year period. All parasite isolates had an IC_50_-ATQ lower than that of the ATQ-resistance C2B reference (IC_50_-ATQ = 13,240 nM), suggesting no ATQ-resistant phenotype. No significant changes in drug susceptibility of DHA, LUM and ATQ were observed during this study period.

**Table 3:** In vitro susceptibility of *P. falciparum* isolates to dihydroartemisinin (DHA), artesunate (AS), artemisone (ATM), chloroquine (CQ), piperaquine (PIP), cycloguanil (CYC), mefloquine (MQ), quinine (QN), lumefantrine (LUM), doxycycline (DOX), atovaquone (ATQ).

**Drug**	**Geometric Mean IC**_**50**_** (nM) (95% CI)**								***P value***
	**W2 clone**		**N**	**2013**	**N**	**2014**	**N**	**2015**	
DHA	3.8 (2.9–4.8)		11	10.8 (8.8–13)	11	7.2 (5.8–8.9)	18	7.1 (5.4–9.5)	0.057
AS	4.8 (3.7–6.1)		11	8.2 (6.7–10)	11	4.8 (3.9–5.9)	18	4.9 (3.8–6.2)	0.005^1,2^
ATM	1.5 (0.8–2.9)		11	1.9 (1.1–3.1)	11	0.7 (0.5–1.0)	18	0.9 (0.6–1.6)	0.021^1^
CQ	234 (189–289)		11	89.3 (42–190)	11	157.4 (101–244)	18	228.4 (145–359)	0.025^2^
PIP	63 (50–78)		11	58.5 (43–79)	10	75.1 (54–104)	16	406.9 (137–1206)	0.006^2^
CYC	3,314 (1,945–5,648)		8	1399 (855–2290)	6	1566 (790–3102)	18	8642 (5086–14,684)	0.0008^2,3^
MQ	62.1 (52–74)		11	18.3 (9.9–34)	11	35.9 (20–63)	18	67.4 (48.9–93.0)	0.0027^2^
QN	277 (232–331)		11	68.2 (42–110)	11	136.8 (99–190)	18	144.9 (113–185)	0.023^2^
LUM	3.6 (2.4–5.4)		11	3.4 (2.3–5.0)	11	6.5 (4.4–9.8)	18	4.7 (3.5–6.4)	0.052
DOX	7,580 (5,858–9,808)		11	23,092 (19,205–27,767)	11	18,491 (15,206–22,485)	18	14,008 (10,737–18,276)	0.025^2^
ATQ	12.5 (7.8–20)		10	1.7 (0.9–3.2)	11	2.5 (1.6–3.8)	17	3.3 (1.8–6.0)	0.253

*P values* calculated by the Kruskal–Wallis test.

^1, 2, 3^Significant difference calculated by Dunn’s multiple comparisons test of data in 2013/2014, 2013/2015, and 2014/2015, respectively.

### In vitro ring stage survival assay (RSA) and *pfk13* mutations

**Figure 2:**
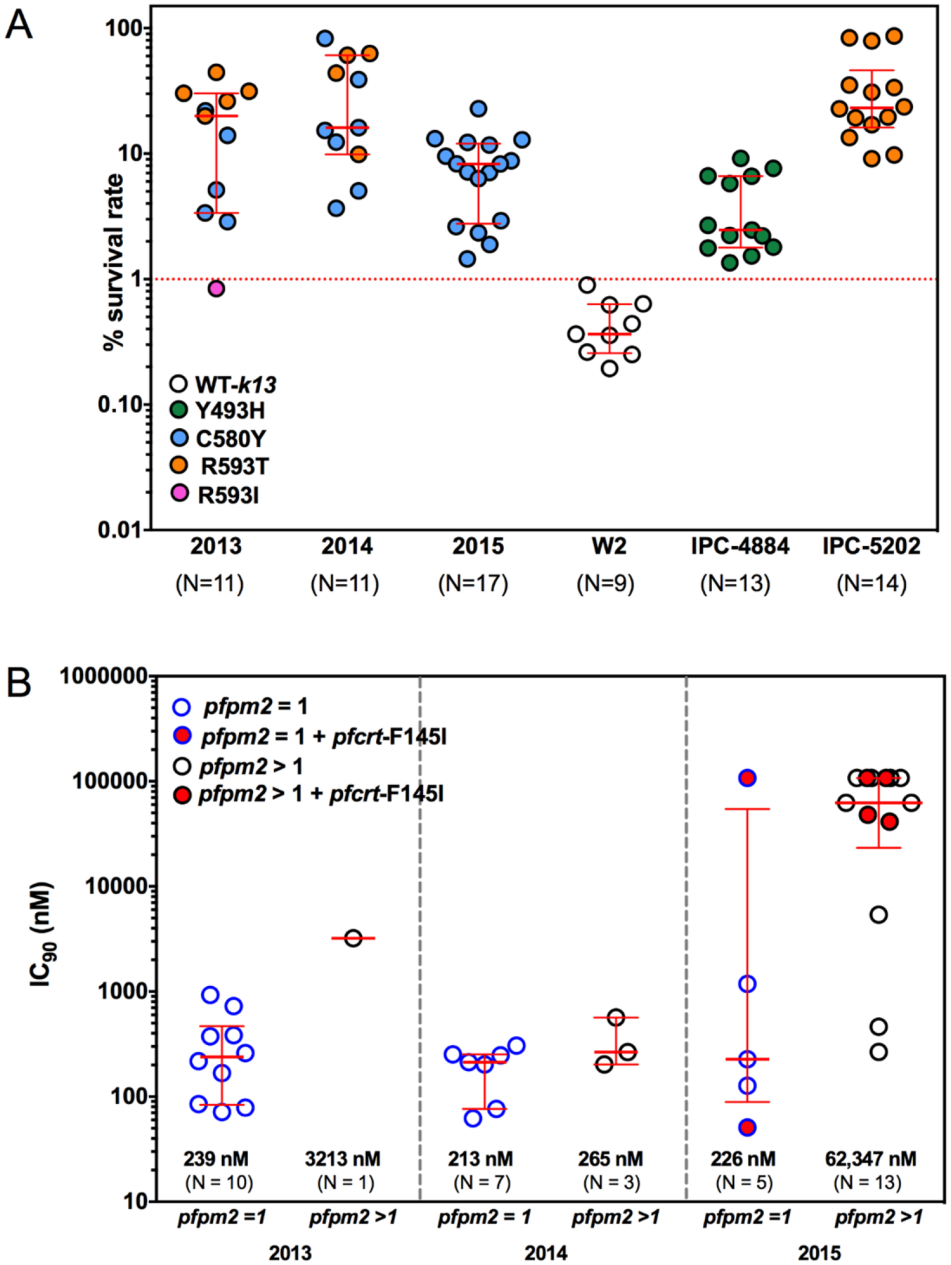
In vitro characterization. (**A**) RSA and *pfk13* mutations. RSA_0-3 h_ survival rate for standard laboratory-adapted clones (W2 for ART-sensitive control, IPC-4884 and IPC-5202 for ART-resistance control) and culture-adapted clinical isolates. The dashed line represents the 1% survival rate cut-off that differentiates ART-resistance (≥ 1% red-dashed line) from ART-sensitive (< 1%) parasites in RSAs. Median and interquartile ranges are shown. (**B**) IC_90_ to PIP in parasites with *pfcrt*-F145I mutation, with and without *pfpm2* amplification. Copy number variations of *pfpm2* are shown on the x-axis, and PIP-IC_90_ is shown on the y-axis (log_10_ scale).

To better understand ART-resistance, 40 isolates from 2013 to 2015 were tested in in vitro RSA_0-3 h_ to measure % survival rate against DHA, and the association with *pfk13* mutations assessed (Fig. 2A). One isolate was excluded due to the growth rate between 0 and 72 h being less than 1.5, leaving 39 isolates evaluable by RSA_0-3 h_. Survival rates for the 11 evaluable isolates from 2013 ranged from 0.84 to 44.5% (median 19.9, IQR of 3.4–30.3), for the 11 evaluable 2014 isolates the range was 3.7 to 82.8% (median 16.1, IQR of 9.9 -60.9) and for the 17 evaluable isolates in 2015 ranged from 1.4 to 22.8% w (median 8.3, IQR of 2.8 -22.8), whereas reference clone survival rate was as expected with medians of 0.4, 2.5, and 23.2% for ART-sensitive W2, ART-resistant IPC-4884 and IPC-5202, respectively. In 2013, 90.9% (10/11) were deemed ART-resistant, all of which contained either *pfk13*-R539T or -C580Y mutations, while the parasite harboring *pfk13*-R539I was sensitive to ART. In 2014 and 2015, all tested parasites containing either *pfk13*-R539T or –C580Y mutation were deemed ART resistant. No significant difference in the proportion of resistant isolates between 2013 and 2014 were found (*P* > 0.05) but there was a significant decrease in median survival rate for 2015 (8.3), compared to those from 2014 (16.1) and 2013 (20.9), Mann–Whitney test, *P* = 0.004 and *P* = 0.02, respectively. It was noted that % survive rate of the parasite harboring *pfk13*-R539I was 0.84%, closer to the cut-off value.

### PIP-susceptibility of parasites with PIP molecular markers

With the emergence of PIP resistance associated with DHA-PIP treatment failures identified in Cambodia from 2010–2013^41^, the distribution of PIP-IC_90_ values for parasites from Thailand (with newly emerged *pfcrt*-F145I mutation with or without amplified *pfpm2*) is displayed in Fig. 2B. In 2013, there was only one isolate with multiple *pfpm2* copies and it had an elevated IC_90_ value. In 2014, no significant differences in IC_90_ values in the isolates with single and multiple *pfpm2* copies were observed (*P* > 0.05). In 2015 there was a significant difference in parasites with single (226 nM) and multiple *pfpm2* copies (62,347 nM) without *pfcrt*-F145I (P = 0.036) whereas no significant differences in IC_90_ values in parasites with single (53,930 nM) and multiple *pfpm2* copies (77,882 nM) with *pfcrt*-F145I (P > 0.05). Isolates with single *pfpm2* and wild type *pfcrt* had more than tenfold lower IC_90_s than parasites with multiple *pfpm2* and/or *pfcrt-*F145I. With the exception of 1 isolate, parasites harboring *pfcrt-*F145I mutation exhibited higher IC_90_ values than those without the mutation (PIP-IC_90_s for standard clone W2, D6 and C2B: 124, 157, and 232 nM, respectively).

In the light of an association between PIP resistance and haplotypes, Table S4 shows PIP-IC_90_s of the parasite isolates in different haplotype subgroups. Parasite isolates containing multiple *pfpm2* copies alone (group III), the *pfcrt*-F145I alone (group V), or the combination of both markers exhibited noticeably high IC_90_ values, indicative of PIP resistance phenotype.

### Preexisting antimalarial treatment

During the enrollment process, participants were queried about recent malaria infection or antimalarial drug use and also past medical history of malaria. Of 112 participants, 27 (24.1%) had reported prior malaria infection: 15 (55.6%), 7 (25.9%), 3 (11.1%), and 2 (7.4) had 1, 2, 3, and 4 episodes of malaria in the last 12 months. Data on antimalarial treatment prior to enrollment in this study revealed that 78 participants (69.6%) had not taken any antimalarial drugs. The most commonly used antimalarials were primaquine (PQ) (26.8%), followed by AS and MQ (16.1%) and CQ (10.7%).

Many antimalarials are long-acting and participants may not disclose use of antimalarial drugs; therefore, antimalarial drug levels in plasma samples were determined by two independent techniques, ex vivo bioassay and LC–MS/MS. In the ex vivo bioassay, 12 of 112 patient plasma samples (10.7%) evaluated against *P. falciparum* W2 clone had significant antimalarial activity (> 17.6 nM DHA equivalents) (Table 4), while 89.3% of evaluable samples were considered negative (≤ 17.6 nM). The positive results were measured in DHA activity equivalents ranging from 49.8 to 3,855 nM with a median activity of 234.2 nM (IQR of 94.3 to 751.4 nM). When LC–MS/MS was employed to detect antimalarial drugs in patient plasma samples, 21 samples (18.8%) were found to contain some antimalarial drugs (Table 4). Sixteen of the 21 had MQ, the first-line therapy for *P. falciparum* at the time of the study and with a half-life of 8 to 20 days. Six had PQ or cPQ; in 2013–2015 a single dose of PQ had not yet been widely used for *P. falciparum* anti-gametocyte activity but it was first-line for *P. vivax* infections. Interestingly four people had evidence of DHA or AS which has a very short half-life (2–3 h). When comparing both methods, 12 patient plasma samples were positive for antimalarial drugs in both the ex vivo bioassay and LC–MS/MS methods while another 9 samples were negative in the ex vivo bioassay but were positive by LC–MS/MS. It is important to note that only 8 samples (38.1%) from the positive samples could be in vitro cultured and evaluated for their drug susceptibility, which showed no significant difference to those without the baseline medicines.

**Table 4:** Comparison of preexisting antimalarial activity in the samples obtained by ex vivo bioassay and liquid chromatography-mass spectrometry (LC–MS/MS).

Sample ID	Ex vivo bioassay (nM DHA equivalents)	Drugs detected by LC–MS
BA-005	79.4	MQ, cMQ
BA-006	90.6	MQ, cMQ
BA-028	105.6	MQ, cMQ
BA-032	–	MQ, cMQ
BA-046	3,855	DHA, MQ
BA-047	–	PQ, cPQ
BA-048	207.5	MQ, cMQ
BA-053	–	PQ. cPQ
BA-055	–	MQ, cMQ
BA-057	49.8	MQ, cMQ
BA-059	260.9	AS
BA-064	758.1	AS,DHA, PQ, cPQ
BA-066	731.3	AS, DHA, MQ
BA-068	–	MQ, cMQ
BA-072	–	PQ, cPQ
BA-078	191.4	MQ, cMQ
BA-084	840.4	DHA,PQ, cPQ, MQ, cMQ
BA-087	468.4	PQ, cPQ, MQ, cMQ
BA-095	–	MQ, cMQ
BA-103	–	MQ, cMQ
BA-110	–	MQ, cMQ

## Discussion

Even though the Greater Mekong Subregion (GMS) has long been the epicenter of antimalarial drug resistance, these countries are aiming to achieve malaria elimination by 2030^49^. Military populations along the Thailand-Cambodia border often patrol in forested areas where the majority of multidrug resistant malaria in this region has been identified. We strategically located our lab at the nearest clinic. Similar approaches of forward deployed laboratories can be applied to migrants and occupations where follow-up post treatment is difficult, including workers in forestry, agriculture or animal husbandry and refugee populations. Despite the limitations of the study, where clinical treatment outcomes were not readily available, we were able to characterize the changes in drug resistance markers overtime in this difficult to reach population.

Chloroquine resistance was first reported in the GMS the 1950s^50^, and several groups^51–55^ have characterized CQ resistant parasite lineages as one of four mutant *pfcrt* genotypes at positions 72–76 (CVIET, SVMNT, CVMNT, and CVMET; mutation underlined). The present results show that all collected parasite isolates harbored the *pfcrt* 72–76 CVIET haplotype similar to previous reports^56–58^. Even though CQ sensitivity was still slightly higher than that of the CQ-resistance W2 reference clone, when the geometric mean IC_50_s of the CQ-sensitive isolates of 30.1 nM^59^ was applied, all the parasites collected under this study are deemed CQ-resistant. Decreased CQ sensitivity was observed from 2013 to 2015 that can likely be attributed to continued CQ use in Thailand for treatment of *P. vivax*.

Parasite isolates carrying the artemisinin resistant gene have been reported in Thailand^58,60^. The current study showed that the *pfk13*-C580Y mutation was predominant, rising from 63% in 2013 to 100% in 2015. These findings are in good agreement with those reported by others^41,58^ in that several *pfk13* mutations developed in Sisaket (62% 580Y)^41^ but by 2015 the C580Y mutation became the predominant mutation found^58^. In contrast, in southern Thailand (Yala Province) very few isolates with *pfk13* mutations have been detected^58,61^. The *pfk13*-R539I found in this study has not yet been reported in Thailand. The *pfk13*-R539I mutation was previously observed at 0.9% in Ghanaian isolates^62^. As assessed by the RSA_0-3 h_, the R539I mutation does not appear to be associated with ART resistance. All the collected isolates had a wild-type cytochrome b gene (*pfcytb*) at both codons 258 and 268 (known to be associated with atovaquone/proguanil treatment failure ^37,39,63^) which is consistent with other isolates collected along Thai-Myanmar and Thai-Cambodia borders during 1998–2005^64^. Of all the *pfmdr1* SNPs analyzed, allelic variation was only observed in *pfmdr1* position 184. This is in good agreement with the previous report by Thita et al.^65^, in which 89% of *P. falciparum* isolates from the Thai-Cambodia border in Chanthaburi and Trat province from 1988 to 2016 had the *pfmdr1* 184F allele. Previous studies in Uganda and Bioko Island suggest that this allele may play a certain role in mediating resistance to some antimalarials^26,66^, and while in the current study, decreased susceptibility to QN, CQ, and MQ was observed, causality cannot be established. Different results were observed from the *P. falciparum* isolates from the southern part of Thailand, with the *pfmdr1* 86Y allele significantly more common^67^.

It was previously reported that parasites containing a pair of point mutations in *P. falciparum* dihydrofolate reductase (*pfdhfr*) (A16V and S108T) are resistant to cycloguanil but not to pyrimethamine, while those with I164L in conjunction with S108N show high levels of resistance to both cycloguanil and pyrimethamine^68^. We did not sequence *pfdhfr* and *P. falciparum* dihydropteroate synthase (*pfdhps*) genes in our study. However, based on previously published data demonstrating fixed proguanil resistance in this region^69,70^, it would be no surprise that parasites in the present study would carry significant antifolate resistance on a background of *pfdhfr-pfdhps* mutations. This is consistent with the study results demonstrating high IC_50_ values for CYC.

In addition to SNPs in established malaria resistance markers, *pfpm2* and *pfmdr1* increased copy numbers are associated with PIP and MQ resistance, respectively. The increased trend in multiple *pfpm2* copy numbers were clearly seen with a decline in multiple *pfmdr1* copy numbers. Novel mutations of the *pfcrt* gene downstream of the 4-aminoquinoline resistance locus (positions 72 to 76), including T93S, H97Y, F145I, I218F, M343L, or G353V, were recently shown to be associated with PIP resistance^31,33,34^. From this study, there was a relatively rapid rise in the F145I mutation, from 0% in 2013 to 30% in 2015. In this study, IC_90_ values of PIP were employed to investigate the PIP resistant phenotypes associated with the tested genetic markers. Parasite isolates holding either multiple *pfpm2* copies with *pfcrt*-F145 or the combination of single or multiple *pfpm2* copy numbers with *pfcrt*-F145I mutation exhibited elevated IC_90_ values of PIP. The high IC_90_ values of parasite isolates with only multiple *pfpm2* copy numbers could be stemmed from *pfpm2* amplification or other novel *pfcrt* mutations that were not detected in this study. Previous studies have shown that the overexpression of *pfpm2* and *pfpm3* in the 3D7 genetic background did not alter PIP sensitivity, suggesting that the increase in *pfpm2* copy number alone is not the sole modulator of PIP resistance^46,71^.

In 2013, an AFRIMS study in Anlong Veng, Cambodia, 12 km from the Cambodia-Thai border at Sisaket province, showed a similar rate of 65% C580Y *pfk13* mutant^40^. When the 54% failure rate of DHA-PIP was observed in Anlong Veng in 2013^40^, the molecular markers for neither ART nor PIP were available so it is only retrospectively that the association of molecular markers and in vitro data can be seen. Similarly in Sisaket, the 87% failure rate of DHA-PIP from the TRACII study (2015–2018)^60^ may have been predicted from this surveillance data.

The use of previous antimalarial drugs or preexisting antimalarial activity in patients can have an effect on malaria treatment^45^. A bioassay method was developed for the measurement of antimalarial activities of ART and its derivatives in either the plasma or sera of patients^72,73^, while bioanalytical LC–MS/MS has widely been used to detect several drugs^74,75^. For example, 3 samples found positively in the LC–MS/MS method contained PQ, the erythrocytic activity of which is little, hence not detected in ex vivo bioassay. Another 6 samples, on the other hand, were found negative in ex vivo bioassay but positive in LC–MS/MS method with the presence of MQ and cMQ at the concentration lower than 150 ng/ml. These results suggest that the bioassay was able to screen blood stage antimalarial activity in certain levels but could not specify which drugs or metabolites are present in the patient plasma samples whereas LC–MS/MS method could. In this study, medical records of the *P. vivax* infected patient were not included in our data set; however, *P. vivax* coinfection is common in this region^70^. It should be noted that PQ would not be expected to persist long in the serum, and the far more likely explanation is that PQ was given as a treatment to prevent transmission of *P. falciparum*. This is now common practice in Thailand.

The difficulty of choosing a national first-line therapy in the GMS is due to differing patterns of antimalarial resistance as we illustrated. High rates of AS-MQ failures along the Thai-Myanmar border^16,76^ resulted in the first-line treatment being changed to DHA-PIP in 2015. However, the parasites from this high risk mobile military population had patterns developed over three years which were more similar to isolates in Cambodia, and then manifested a similar drop in DHA-PIP efficacy. The Thai government responded to that regional challenge in 2019, by changing the first-line treatment for *P. falciparum* to artesunate-pyronaridine (AS-PND) in Sisaket and Ubon Ratchathani Provinces. Recent reports suggest there are two lineages within the GMS. The C580Y mutation predominate in the eastern GMS and the F466I mutation has spread along the Myanmar border^58^. While there may be predominant lineages which have emerged, continued local surveillance will be important to determine if and how these patterns shift. The emergence of the novel *pfcrt-*F145I mutation in 2014 from this study followed the emergence and DHA-PIP treatment failures in Cambodia^40,45,77^. Shrestha, et al*.*^78^ showed that more than 98% of the parasites collected from northern Cambodia carried newly emerged *pfcrt* mutations and after 2014, the prevalence of parasites with *pfcrt*-F145I mutation started to decrease, being out-competed by other less resistant, but more fit *pfcrt* variants such as *pfcrt*-T93S and I218F^34^. Targeting surveillance studies on more mobile human populations along border areas such migrants and militaries, can help predict drug efficacy or first detect any introduction of novel mutations which may interrupt the path to malaria elimination in the GMS.

A few key limitations of this study should be considered. This includes the fact that the study samples were collected more than 5 years ago from one geographical area. Therefore, drug resistance profiles reported here should not be construed for all of Thailand. Due to inability to culture adapt all the samples, only 33% of the collected samples could be analyzed for in vitro drug susceptibility. The majority of the samples were genotyped for the presence of drug resistance SNPs; however, it is realized that other SNPs not included in this study may contribute to drug resistance. Finally, multiplicity of infections was not examined. However, future studies will employ whole genome sequencing to address the complexity of multiple infections.

## Supplementary Information

Supplementary Information.

## Data Availability

All data generated or analyzed during this study are included in this published article and its supplementary information files.

## References

[1] World Malaria Report. (World Health Organization, Geneva, 2020).

[2] Luxemburger C (1998). Two patients with falciparum malaria and poor in vivo responses to artesunate. Trans. R. Soc. Trop. Med. Hyg..

[3] Wongsrichanalai C, Meshnick SR (2008). Declining artesunate-mefloquine efficacy against falciparum malaria on the Cambodia-Thailand border. Emerg. Infect. Dis..

[4] Noedl H (2008). Evidence of artemisinin-resistant malaria in western Cambodia. N. Engl. J. Med..

[5] Dondorp AM (2009). Artemisinin resistance in *Plasmodium falciparum* malaria. N. Engl. J. Med..

[6] Amaratunga C (2012). Artemisinin-resistant *Plasmodium falciparum* in Pursat province, western Cambodia: A parasite clearance rate study. Lancet. Infect. Dis..

[7] Phyo AP (2012). Emergence of artemisinin-resistant malaria on the western border of Thailand: A longitudinal study. Lancet.

[8] Kyaw MP (2013). Reduced susceptibility of *Plasmodium falciparum* to artesunate in southern Myanmar. PLoS ONE.

[9] Ashley EA (2014). Spread of artemisinin resistance in *Plasmodium falciparum* malaria. N. Engl. J. Med..

[10] Ariey F (2014). A molecular marker of artemisinin-resistant *Plasmodium falciparum* malaria. Nature.

[11] Ghorbal M (2014). Genome editing in the human malaria parasite *Plasmodium falciparum* using the CRISPR-Cas9 system. Nat. Biotechnol..

[12] Fairhurst RM (2015). Understanding artemisinin-resistant malaria: What a difference a year makes. Curr. Opin. Infect. Dis..

[13] Straimer J (2015). Drug resistance. K13-propeller mutations confer artemisinin resistance in *Plasmodium falciparum* clinical isolates. Science.

[14] Witkowski B (2013). Novel phenotypic assays for the detection of artemisinin-resistant *Plasmodium falciparum* malaria in Cambodia: In-vitro and ex-vivo drug-response studies. Lancet. Infect. Dis.

[15] Price RN (2004). Mefloquine resistance in *Plasmodium falciparum* and increased pfmdr1 gene copy number. Lancet.

[16] Phyo AP (2016). Declining efficacy of artemisinin combination therapy against *P. falciparum* malaria on the Thai-Myanmar Border (2003–2013): The role of parasite genetic factors. Clin. Infect. Dis..

[17] Eyase FL (2013). The role of Pfmdr1 and Pfcrt in changing chloroquine, amodiaquine, mefloquine and lumefantrine susceptibility in western-Kenya *P. falciparum* samples during 2008–2011. PLoS ONE.

[18] Foote SJ (1990). Several alleles of the multidrug-resistance gene are closely linked to chloroquine resistance in Plasmodium falciparum. Nature.

[19] Povoa MM (1998). Pfmdr1 Asn1042Asp and Asp1246Tyr polymorphisms, thought to be associated with chloroquine resistance, are present in chloroquine-resistant and -sensitive Brazilian field isolates of Plasmodium falciparum. Exp. Parasitol..

[20] Sidhu AB, Valderramos SG, Fidock DA (2005). pfmdr1 mutations contribute to quinine resistance and enhance mefloquine and artemisinin sensitivity in Plasmodium falciparum. Mol. Microbiol..

[21] Preechapornkul P (2009). Plasmodium falciparum pfmdr1 amplification, mefloquine resistance, and parasite fitness. Antimicrob. Agents Chemother..

[22] Borges S (2011). Genomewide scan reveals amplification of mdr1 as a common denominator of resistance to mefloquine, lumefantrine, and artemisinin in Plasmodium chabaudi malaria parasites. Antimicrob. Agents Chemother..

[23] Lekana-Douki JB (2011). Increased prevalence of the Plasmodium falciparum Pfmdr1 86N genotype among field isolates from Franceville, Gabon after replacement of chloroquine by artemether-lumefantrine and artesunate-mefloquine. Infect. Genet. Evol..

[24] Lopes D (2002). Molecular characterisation of drug-resistant Plasmodium falciparum from Thailand. Malar. J..

[25] Wurtz N (2014). Role of Pfmdr1 in in vitro Plasmodium falciparum susceptibility to chloroquine, quinine, monodesethylamodiaquine, mefloquine, lumefantrine, and dihydroartemisinin. Antimicrob. Agents Chemother..

[26] Dokomajilar C, Nsobya SL, Greenhouse B, Rosenthal PJ, Dorsey G (2006). Selection of Plasmodium falciparum pfmdr1 alleles following therapy with artemether-lumefantrine in an area of Uganda where malaria is highly endemic. Antimicrob. Agents Chemother..

[27] Amato R (2017). Genetic markers associated with dihydroartemisinin-piperaquine failure in Plasmodium falciparum malaria in Cambodia: a genotype-phenotype association study. Lancet. Infect. Dis.

[28] Parobek CM (2017). Partner-drug resistance and population substructuring of artemisinin-resistant Plasmodium falciparum in Cambodia. Genome Biol. Evol..

[29] Witkowski B (2017). A surrogate marker of piperaquine-resistant Plasmodium falciparum malaria: A phenotype-genotype association study. Lancet. Infect. Dis..

[30] Bopp S (2018). Plasmepsin II-III copy number accounts for bimodal piperaquine resistance among Cambodian Plasmodium falciparum. Nat. Commun..

[31] Duru V (2015). Plasmodium falciparum dihydroartemisinin-piperaquine failures in Cambodia are associated with mutant K13 parasites presenting high survival rates in novel piperaquine in vitro assays: retrospective and prospective investigations. BMC Med..

[32] Agrawal S (2017). Association of a novel mutation in the plasmodium falciparum chloroquine resistance transporter with decreased piperaquine sensitivity. J. Infect. Dis..

[33] Ross LS (2018). Emerging Southeast Asian PfCRT mutations confer Plasmodium falciparum resistance to the first-line antimalarial piperaquine. Nat. Commun..

[34] Dhingra SK, Small-Saunders JL, Menard D, Fidock DA (2019). Plasmodium falciparum resistance to piperaquine driven by PfCRT. Lancet. Infect. Dis.

[35] Thanh NV (2017). Rapid decline in the susceptibility of Plasmodium falciparum to dihydroartemisinin-piperaquine in the south of Vietnam. Malar. J..

[36] Chaorattanakawee S (2016). Ex vivo piperaquine resistance developed rapidly in Plasmodium falciparum isolates in northern Cambodia compared to Thailand. Malar. J..

[37] Korsinczky M (2000). Mutations in Plasmodium falciparum cytochrome b that are associated with atovaquone resistance are located at a putative drug-binding site. Antimicrob. Agents Chemother..

[38] Schwobel B, Alifrangis M, Salanti A, Jelinek T (2003). Different mutation patterns of atovaquone resistance to Plasmodium falciparum in vitro and in vivo: rapid detection of codon 268 polymorphisms in the cytochrome b as potential in vivo resistance marker. Malar. J..

[39] Plucinski MM (2014). Novel mutation in cytochrome B of *Plasmodium falciparum* in one of two atovaquone-proguanil treatment failures in travelers returning from same site in Nigeria. Open Forum Infect. Dis..

[40] Spring MD (2015). Dihydroartemisinin-piperaquine failure associated with a triple mutant including kelch13 C580Y in Cambodia: An observational cohort study. Lancet. Infect. Dis..

[41] Talundzic E (2015). Selection and spread of artemisinin-resistant alleles in Thailand prior to the global artemisinin resistance containment campaign. PLoS Pathog..

[42] Dow GS, Gettayacamin M, Hansukjariya P, Imerbsin R, Komcharoen S, Sattabongkot J, Kyle D, Mihous W, Cozen S, Kenworthy D, Miller A, Veazey J, Ohrt C (2011). Radical curative efficacy of tafenoquine combination regimens in *Plasmodium cynomolgi*-infected Rhesus monkeys (Macaca mulatta). Malar. J..

[43] Trager W, Jensen JB (1976). Human malaria parasites in continuous culture. Science.

[44] Chaorattanakawee S (2013). Direct comparison of the histidine-rich protein-2 enzyme-linked immunosorbent assay (HRP-2 ELISA) and malaria SYBR green I fluorescence (MSF) drug sensitivity tests in Plasmodium falciparum reference clones and fresh ex vivo field isolates from Cambodia. Malar. J..

[45] Chaorattanakawee S (2015). Ex vivo drug susceptibility testing and molecular profiling of clinical *Plasmodium falciparum* isolates from Cambodia from 2008 to 2013 suggest emerging piperaquine resistance. Antimicrob. Agents Chemother..

[46] Boonyalai N (2020). Piperaquine resistant Cambodian Plasmodium falciparum clinical isolates: In vitro genotypic and phenotypic characterization. Malar. J..

[47] Noedl H, Teja-Isavadharm P, Miller RS (2004). Nonisotopic, semiautomated plasmodium falciparum bioassay for measurement of antimalarial drug levels in serum or plasma. Antimicrob. Agents Chemother..

[48] Teja-Isavadharm P (2004). Plasmodium falciparum-based bioassay for measurement of artemisinin derivatives in plasma or serum. Antimicrob. Agents Chemother..

[49] Strategy for malaria elimination in the GMS (2015–2030). (World Health Organization, 2015).

[50] Ridley RG (2002). Medical need, scientific opportunity and the drive for antimalarial drugs. Nature.

[51] Wellems TE (1990). Chloroquine resistance not linked to mdr-like genes in a Plasmodium falciparum cross. Nature.

[52] Wellems TE, Walker-Jonah A, Panton LJ (1991). Genetic mapping of the chloroquine-resistance locus on Plasmodium falciparum chromosome 7. Proc. Natl. Acad. Sci. U.S.A..

[53] Su X, Kirkman LA, Fujioka H, Wellems TE (1997). Complex polymorphisms in an approximately 330 kDa protein are linked to chloroquine-resistant P. falciparum in Southeast Asia and Africa. Cell.

[54] Fidock DA (2000). Mutations in the *P. falciparum* digestive vacuole transmembrane protein PfCRT and evidence for their role in chloroquine resistance. Mol. Cell.

[55] Sidhu AB, Verdier-Pinard D, Fidock DA (2002). Chloroquine resistance in *Plasmodium falciparum* malaria parasites conferred by pfcrt mutations. Science.

[56] Takahashi N (2012). Large-scale survey for novel genotypes of *Plasmodium falciparum* chloroquine-resistance gene pfcrt. Malar. J..

[57] Buppan P (2018). Multiple novel mutations in *Plasmodium falciparum* chloroquine resistance transporter gene during implementation of artemisinin combination therapy in Thailand. Am. J. Trop. Med. Hyg..

[58] Imwong M (2020). Molecular epidemiology of resistance to antimalarial drugs in the Greater Mekong subregion: An observational study. Lancet. Infect. Dis.

[59] Bickii J, Basco LK, Ringwald P (1998). Assessment of three in vitro tests and an in vivo test for chloroquine resistance in *Plasmodium falciparum* clinical isolates. J. Clin. Microbiol..

[60] van der Pluijm RW (2019). Determinants of dihydroartemisinin-piperaquine treatment failure in *Plasmodium falciparum* malaria in Cambodia, Thailand, and Vietnam: a prospective clinical, pharmacological, and genetic study. Lancet. Infect. Dis..

[61] Noisang C (2019). Molecular detection of drug resistant malaria in Southern Thailand. Malar. J..

[62] Matrevi SA (2019). Plasmodium falciparum Kelch propeller polymorphisms in clinical isolates from Ghana from 2007 to 2016. Antimicrob. Agents Chemother..

[63] Fivelman QL, Butcher GA, Adagu IS, Warhurst DC, Pasvol G (2002). Malarone treatment failure and in vitro confirmation of resistance of Plasmodium falciparum isolate from Lagos, Nigeria. Malar. J..

[64] Khositnithikul R, Tan-Ariya P, Mungthin M (2008). In vitro atovaquone/proguanil susceptibility and characterization of the cytochrome b gene of Plasmodium falciparum from different endemic regions of Thailand. Malar. J..

[65] Thita T (2018). Phenotypic and genotypic characterization of Thai isolates of Plasmodium falciparum after an artemisinin resistance containment project. Malar. J..

[66] Li J (2014). High prevalence of pfmdr1 N86Y and Y184F mutations in Plasmodium falciparum isolates from Bioko Island Equatorial Guinea. Pathogens Glob. Health.

[67] Mungthin M (2014). Distribution of pfmdr1 polymorphisms in Plasmodium falciparum isolated from Southern Thailand. Malar. J..

[68] Peterson DS, Milhous WK, Wellems TE (1990). Molecular basis of differential resistance to cycloguanil and pyrimethamine in Plasmodium falciparum malaria. Proc. Natl. Acad. Sci. U.S.A..

[69] Cheychom J, Kanchanakhan N, Vijaykadga S, Harnyuttanakorn P (2013). Antifolate resistance mutation and proguanil susceptibility among *Plasmodium falciparum* isoaltes in Thai-Cambodia border. J. Health Res..

[70] Wojnarski, M. *et al.* Atovaquone-Proguanil in Combination With Artesunate to Treat Multidrug-Resistant P. falciparum Malaria in Cambodia: An Open-Label Randomized Trial. *Open Forum Infect Dis* **6**, ofz314, doi:10.1093/ofid/ofz314 (2019). 10.1093/ofid/ofz314 PMC6736354 31660398

[71] Loesbanluechai D (2018). Overexpression of plasmepsin II and plasmepsin III does not directly cause reduction in Plasmodium falciparum sensitivity to artesunate, chloroquine and piperaquine. Int. J. Parasitol. Drugs Drug Resist.

[72] Traore B, Lazaro E, Gay F (1997). A bioassay for evaluating antimalarial activity and for measuring concentration in plasma. Trop. Med. Int. Health TM & IH.

[73] Ittarat W (1998). Effects of alpha-thalassemia on pharmacokinetics of the antimalarial agent artesunate. Antimicrob. Agents Chemother..

[74] Chhonker YS, Edi C, Murry DJ (2018). LC-MS/MS method for simultaneous determination of diethylcarbamazine, albendazole and albendazole metabolites in human plasma: Application to a clinical pharmacokinetic study. J. Pharm. Biomed. Anal..

[75] Kaewkhao K (2019). High sensitivity methods to quantify chloroquine and its metabolite in human blood samples using LC-MS/MS. Bioanalysis.

[76] Carrara VI (2009). Changes in the treatment responses to artesunate-mefloquine on the northwestern border of Thailand during 13 years of continuous deployment. PLoS ONE.

[77] Saunders DL (2014). Dihydroartemisinin-piperaquine failure in Cambodia. N. Engl. J. Med..

[78] Shrestha, B. *et al.* Distribution and temporal dynamics of P. falciparum chloroquine resistance transporter mutations associated with piperaquine resistance in Northern Cambodia. *J. Infect. Dis.* doi:10.1093/infdis/jiab055(2021). 10.1093/infdis/jiab055 PMC8448439 33528566

