# Supplementary material for: Plasmodium falciparum phenotypic and genotypic resistance profile during the emergence of Piperaquine resistance in Northeastern Thailand
Source: Sci Rep. 2021 Jun 28;11:13419. doi: 10.1038/s41598-021-92735-6 (PMC8238947; doi:10.1038/s41598-021-92735-6)
Supplement: Supplementary file 1 — Supplementary Information. [file 41598_2021_92735_MOESM1_ESM.docx]

**Supporting Information**

***Plasmodium falciparum* phenotypic and genotypic resistance profile during the emergence of Piperaquine resistance in Northeastern Thailand**

Nonlawat Boonyalai^1^*, Chatchadaporn Thamnurak^1^, Piyaporn Sai-ngam^1^, Winita Ta-aksorn^1^, Montri Arsanok^1^, Nichapat Uthaimongkol^1^, Siratchana Sundrakes^1^, Sorayut Chattrakarn^1^, Chaiyaporn Chaisatit^1^, Chantida Praditpol^1^, Watcharintorn Fagnark^1^, Kirakarn Kirativanich^1^, Suwanna Chaorattanakawee^1,2^, Pattaraporn Vanachayangkul^1^, Paphavee Lertsethtakarn^1^, Panita Gosi^1^, Darunee Utainnam^6^, Wuttikon Rodkvamtook^6^, Worachet Kuntawunginn^1^, Brian A. Vesely^1^, Michele D. Spring^1^, Mark M. Fukuda^3^, Charlotte Lanteri^5^, Douglas Walsh^7^, David L. Saunders^4^, Philip L. Smith^5^, Mariusz Wojnarski^1^, Narongrid Sirisopana^6^, Norman C. Waters^1^, Krisada Jongsakul^1^, Jariyanart Gaywee^6^.

^1^Department of Bacterial and Parasitic Diseases, Armed Forces Research Institute of Medical Sciences, Bangkok, Thailand.

^2^Department of Parasitology and Entomology, Faculty of Public Health, Mahidol University, Bangkok, Thailand.

^3^Department of Retrovirology, Armed Forces Research Institute of Medical Sciences, Bangkok, Thailand.

^4^U.S. Army Research Institute of Infectious Diseases, Frederick, MD, United States.

^5^Walter Reed Army Institute of Research. Silver Spring, Maryland 20910.

^6^ Royal Thai Army Component, Armed Forces Research Institute of Medical Sciences, Bangkok, Thailand.

^7^Department of Dermatology, Syracuse VA medical center.

Short running title: *P. falciparum* resistance profile in Thai-Cambodia border during the emergence of Piperaquine resistance.

*Correspondence: Nonlawat Boonyalai, [nonlwatb.fsn@afrims.org](mailto:nonlwatb.fsn@afrims.org)

**Table S1**. Sequences of *P. falciparum* primers for drug-resistant markers including *pfkelch13* propeller domain, *pfcrt* (amino acid position 145), and *pfbc1* (amino acid positions 258, and 268).

| **Primer** | | **Sequence (5´ 🡪 3´)** | | **Reference** |
| --- | --- | --- | --- | --- |
| *pfkelch13* | Outer Forward | | CGG AGT GAC CAA ATC TGG GA |  |
|  | Outer Reverse | | GGG AAT CTG GTG GTA ACA GC | https://www.wwarn.org/tools-resources/procedures/pcr-and-sequencing-genotyping-candidate-plasmodium-falciparum-artemisinin |
|  | Nested Forward | | GCC AAG CTG CCA TTC ATT TG |  |
|  | Nested Reverse | | GCC TTG TTG AAA GAA GCA GA |  |
| *pfcrt* | Forward | | GAACGACACCGAAGCTTTA | In-house method |
|  | Reverse | | GATTGGATATTTCCAGTAGTTCTTG |  |
| *pfcytb* | Forward | | TGGTAGCACAAATCCTTTAGGG | In-house method |
|  | Reverse | | TGTGGTAATTGACATCCAATCC |  |

**Table S2**. Sequences of primers for the detection of *pfcrt* mutation at amino acid positions 72-76 , copy number variation of *pfmdr1* and *pfpm2* with β-tubulin and *pfmdr1* (amino acid positions 86, 184, 1034, and 1042).

| **qPCR** | **Primer** | | **Sequence (5′ 🡪 3′)** | | **Reference** |  |
| --- | --- | --- | --- | --- | --- | --- |
| *pfmdr1* | *pfmdr1* Forward | | | TGC ATC TAT AAA ACG ATC AGA CAA A | [1] |  |
|  | *pfmdr1* Reverse | | | TCG TGT GTT CCA TGT GAC TGT |  |  |
|  | *pfmdr1-*probe | | | FAM-TTT AAT AAC CCT GAT CGA AAT GGA ACC TTT G-TAMRA |  |  |
|  | β-*tubulin* Forward | | | AAA AAT ATG ATG TGC GCA AGT GA |  |  |
|  | β-*tubulin* Reverse | | | AAC TTC CTT TGT GGA CAT TCT TCC T |  |  |
|  | β-*tubulin-*probe | | | VIC-TAG CAC ATG CCG TTA AAT ATC TTC CAT GTC T-TAMRA |  |  |
| *pfpm2* | *pfpm2* Forward | | | TGG TGA TGC AGA AGT TGG AG | [2] |  |
|  | *pfpm2* Reverse | | | TGG GAC CCA TAA ATT AGC AGA |  |  |
|  | β-*tubulin* Forward | | | TGA TGT GCG CAA GTG ATC C |  |  |
|  | β-*tubulin* reverse | | | TCC TTT GTG GAC ATT CTT CCT C |  |  |
| *pfcrt* | *pfcrt* Forward | | | TGG TAA ATG TGC TCA TGT GTT T | [3] |  |
|  | *pfcrt* Revese | | | AGT TTC GGA TGT TAC AAA ACT ATA GT |  |  |
|  | *pfcrt-*CVMNK WT Probe | | | FAM-TGT GTA ATG AAT AAA ATT TTT GCT AA-BHQ1 |  |  |
|  | | *pfcrt-*CVIET Probe | | JOE-TGT GTA ATT GAA ACA ATT TTT GCT AA-BHQ1 | | |
|  | | *pfcrt-*SVMNT Probe | | ROX-AGT GTA ATG AAT ACA ATT TTT GCT AA-BHQ2 | | |
| *pfmdr1*  86  184  1034  1042 | | *Pfmdr1-*Forward  *Pfmdr1-*Reverse  *Pfmdr1-WT* Probe  *Pfmdr1-MT* Probe  *Pfmdr1-*Forward  *Pfmdr1-*Reverse  *Pfmdr1-WT* Probe  *Pfmdr1-MT* Probe  *Pfmdr1-*Forward  *Pfmdr1-*Reverse  *Pfmdr1-WT* Probe  *Pfmdr1-MT* Probe  *Pfmdr1-*Forward  *Pfmdr1-*Reverse  *Pfmdr1-WT* Probe  *Pfmdr1-MT* Probe | | TGT ATG TGC TGT ATT ATC AGG AGG AAC  AAT TGT ACT AAA CCT ATA GAT ACT AAT GAT AAT  ATT ATA GG  6FAM- ACC TAA ATT CAT GTT CTTTT-MGB-NFQ  TET- ACC TAA ATA CAT GTT CTT T-MGB-NFQ  AAG ATG GAC AAT TTC ATG ATA ATA ATC CT  AAT ACA TAA AGT CAA ACG TGC ATT TTT TA  6FAM-CTT TTT AGG TTT ATA TAT TTG GT-MGB-NFQ [4].  TET-CTT TTT AGG TTT ATT TAT TTG GT-MGB-NFQ  AAA AAG AAG AAT TAT TGT AAA TGC AGC TT  GGA TCC AAA CCA ATA GGC AAA A  6FAM-ATT CAG TCA AAG CGC T-MGB-NFQ  TET-ATT CTG TCA AAG CGC T-MGB-NFQ  AAA AAG AAG AAT TAT TGT AAA TGC AGC TT  TTT CCA GCA TAA CTA CCA GTA AAT ATA AAA G  6FAM-CAA TTA TTT ATT AAT AGT TTT GC-MGB-NFQ  TET-AAT TAT TTA TTG ATA GTT TTG C-MGB-NFQ | | |

**REFERENCES**

[1] Price RN, Uhlemann AC, Brockman A, McGready R, Ashley E, Phaipun L, et al. Mefloquine resistance in *Plasmodium falciparum* and increased pfmdr1 gene copy number. Lancet. 2004;364:438-47.

[2] Witkowski B, Duru V, Khim N, Ross LS, Saintpierre B, Beghain J, et al. A surrogate marker of piperaquine-resistant *Plasmodium falciparum* malaria: a phenotype-genotype association study. The Lancet Infectious diseases. 2017;17:174-83.

[3] Sutherland CJ, Haustein T, Gadalla N, Armstrong M, Doherty JF, Chiodini PL. Chloroquine-resistant *Plasmodium falciparum* infections among UK travellers returning with malaria after chloroquine prophylaxis. The Journal of antimicrobial chemotherapy. 2007;59:1197-9.

[4] Purfield A, Nelson A, Laoboonchai A, Congpuong K, McDaniel P, Miller RS, et al. A new method for detection of pfmdr1 mutations in *Plasmodium falciparum* DNA using real-time PCR. Malar J. 2004;3:9.

**Table S3.** Haplotypes and Copy number variation (CNV) of *P. falciparum* isolates collected from 2013 to 2015

| **Group** | **Haplotype** |  |  |  |  | |  |  | **CNV** | | **Total** | **2013** | **2014** | **2015** |
| --- | --- | --- | --- | --- | --- | --- | --- | --- | --- | --- | --- | --- | --- | --- |
|  | ***pfmdr1*** |  | ***pfk13*** |  |  | ***pfcrt*** | |  | ***pfmdr1*** | ***pfpm2*** | **N (%)** | **N (%)** | **N (%)** | **N (%)** |
|  | **Y184F** |  | **R539T/I** | **C580Y** |  | **F145I** | |  |  |  | **112 (100.0)** | **27 (100.0)** | **39 (100.0)** | **46 (100.0)** |
| I | Y184 |  | R539 | 580Y |  | F145 | |  | single | single | 3 (2.7) | 0 (0.0) | 3 (7.7) | 0 (0.0) |
| II | 184F |  | R539 | 580Y |  | F145 | |  | single | single | 27 (24.1) | 15 (55.6) | 6 (15.4) | 6 (13.0) |
| III | 184F |  | R539 | 580Y |  | F145 | |  | single | multiple | 45 (40.2) | 2 (7.4) | 17 (43.6) | 26 (56.5) |
| IV | 184F |  | R539 | 580Y |  | F145 | |  | multiple | single | 1 (0.9) | 0 (0.0) | 1 (2.6) | 0 (0.0) |
| V | 184F |  | R539 | 580Y |  | 145I | |  | single | single | 6 (5.4) | 0 (0.0) | 2 (5.1) | 4 (8.7) |
| VI | 184F |  | R539 | 580Y |  | 145I | |  | single | multiple | 13 (11.6) | 0 (0.0) | 3 (7.7) | 10 (21.7) |
| VII | 184F |  | 539I | C580 |  | F145 | |  | single | single | 1 (0.9) | 1 (3.7) | 0 (0.0) | 0 (0.0) |
| VIII | 184F |  | 539T | C580 |  | F145 | |  | single | single | 12 (10.7) | 6 (22.2) | 6 (15.4) | 0 (0.0) |
| IX | 184F |  | 539T | C580 |  | F145 | |  | multiple | single | 4 (3.6) | 3 (11.1) | 1 (2.6) | 0 (0.0) |

**Table S4.** PIP-IC_90_ and different haplotype subgroups of *P. falciparum* isolates

| **Group** | **N (%)** | **PIP-IC_90_ (nM)** |
| --- | --- | --- |
|  | 40 (100) | **median (IQR)** |
| I | 2 (5) | 233 (213-252) |
| II | 8 (20) | 197 (95-791) |
| III | 14 (35) | 5,376 (364-107,810) |
| IV | 0 | N.D^*^ |
| V | 2 (5) | 428,404 (51-856,758) |
| VI | 4 (10) | 77,882 (42,968-107,810) |
| VII | 1 (3) | 79 |
| VIII | 7 (18) | 260 (217-375) |
| IX | 2 (5) | 137 (71-203) |

^*^N.D = Not determined
